# Nutrition of Shade-Grown Coffee Plantations with Inorganic Fertilizers in Oaxaca, Mexico

**DOI:** 10.3390/plants15081210

**Published:** 2026-04-15

**Authors:** Miguel A. Cano-García, Verónica Mariles-Flores, Patricio Sánchez-Guzmán, Luis E. García-Mayoral, Rafael Ariza-Flores, Pedro Cadena-Iñiguez, Luis A. Gálvez-Marroquín

**Affiliations:** 1 National Institute for Forestry, Agriculture and Livestock Research (INIFAP), Mexico City 04010, Mexico; cano.miguel@inifap.gob.mx (M.A.C.-G.); garcia.eduardo@inifap.gob.mx (L.E.G.-M.); ariza.rafael@inifap.gob.mx (R.A.-F.); cadena.pedro@inifap.gob.mx (P.C.-I.); galvez.luis@inifap.gob.mx (L.A.G.-M.); 2 Postgraduate College, Campus Montecillo, Texcoco 56230, Mexico; sanchezp@colpos.mx

**Keywords:** *Coffea arabica*, inorganic fertilizers, nitrogen, phosphorus, potassium, dolomitic lime, Typica, Oro azteca, agroforestry system, coffee yield

## Abstract

Coffee (*Coffea arabica*) is a very important world commodity because of the countries involved in its production, along with the total cultivated area, production volume, consumption and economic impact. In Mexico, the coffee producing areas are located mainly in the hilly terrain of southern Mexico under agroforestry systems predominantly owned by smallholders. Low productivity is faced especially in the state of Oaxaca as a result of inadequate management practices such as aged plantations and deficient practices of pruning and plant nutrition. In order to evaluate the effect of inorganic fertilization on coffee yield, an experiment was carried out at three plantations located in the coastal coffee producing region of the state of Oaxaca, Mexico. Six treatments considering varied amounts of inorganic nitrogen (N), phosphorus (P) and potassium (K) and lime application were applied in coffee plantations with the varieties Typica and Oro azteca. A randomized complete block design with four replications was used. The experiments were conducted in areas with three- or four-year-old plants, with the objective of having at least one harvest for yield evaluation. The variables’ plant height and coffee yield per plant were registered. The soil was classified based on soil profile description and lab analyses. The results showed that the soil in the study area is a Lithic Ustorthent with low pedogenic evolution and the application of inorganic nitrogen, phosphorus and potassium along with dolomitic lime, increased coffee yield on both varieties of arabica coffee: Typica and Oro azteca.

## 1. Introduction

Coffee is a key internationally traded commodity, with coffee plantations in over 50 countries and about 11 million hectares managed under varied conditions and production systems resulting in productivity levels that range from 2652 kg per hectare to 18 kg per hectare [1]. High productivity is generally obtained in monoculture intensively managed systems [2] while the medium to low productivity is achieved mainly by smallholder farmers with coffee growing under shade trees and with low external input [3]. Coffee world production in the marketing year (MY) 2024/2025 was 100.2 million bags (60 kg) of *C. arabica* and 75.1 million bags of *C. canephora* with Brazil and Colombia as the main *C. arabica* producers, followed by Ethiopia, Honduras, Peru, Mexico and Guatemala. Mexico’s production in this fiscal year (FY) was 3.87 million bags with 91% corresponding to *C. arabica* [4].

Coffee production in Mexico in 2024 was carried out in 701,335 ha mainly in the southern states of Chiapas (34.8%), Veracruz (20.7%), Oaxaca (18.9%) and Puebla (10.2%). These states comprise 84.5% of the total area, 91.4% of the total production and 89.7% of the production value. The remaining production was carried out in the states of Puebla, Guerrero, Hidalgo, San Luis Potosí, Jalisco, Nayarit, Colima, Tabasco, Estado de Mexico, Queretaro and Morelos. The total land area cultivated with coffee in the Oaxaca state in 2024 was 132,268 ha with an average yield of 0.8 t ha^−1^ and a total production volume of 90,257 t of cherry coffee. The lowest cherry coffee yield occurs in the coastal region of Oaxaca with 0.5 t ha^−1^ [5,6].

The production coffee system in Mexico is managed mostly by smallholder farmers, with coffee plantations growing on hilly areas with forest trees providing shade to the coffee plants. The majority of these farmers lack financial resources to invest in methods of improving yields and, as a consequence, cherry coffee yield ranges between 0.4 and 1.7 t ha^−1^, while the national average is 1.6 t ha^−1^ [5].

The low productivity of the coffee system in the state of Oaxaca is a result of several factors such as plantations of seven years or older, few pruning activities and inadequate plant nutrition practices. Because of the lack of technical recommendations and limited investment resources, fertilization, either mineral or organic, is deficient or non-existent. Evidence of the lack of updated research is that the last technical fertilization recommendation was generated by the Instituto Mexicano del Café (INMECAFE), which recommended applying 18-12-00 of N-P-K per hectare [7]. Since the dissolution of INMECAFE in 1989 research institutions have focused primarily on the evaluation of which plant varieties offer the greater resistance to coffee leaf rust, a highly problematic fungus.

Proper coffee fertilization is very important in obtaining adequate bean size and quality, both of which directly affect overall productivity [8]. Nutrients applied replenish those used by the plant to form tissue and fruits as well as those lost due to leaching and immobilization [9]. Coffee plants require high nitrogen and potassium and less phosphorus. Harvesting a hectare from a high yield coffee plantation may extract from the soil, annually, about 135, 34 and 145 kg of nitrogen, phosphorus and potassium, respectively [10].

Knowledge of soil nutrient conditions is essential when designing fertilization rates, especially in hilly areas with high slope values with very low soil organic matter content below the upper horizon. Soil properties in a coffee producing area in southern Mexico with slope values higher than 50% showed medium to high content of organic matter and nitrogen at the surface horizon (0–30 cm depth) but at the subsurface horizons the content of these nutrients decreased drastically [11]. Because of the high organic matter and N content at the soil surface, sampling soil only at the surface horizons may lead to inaccurate conclusions about the soil fertility [12,13].

Research on mineral fertilization of coffee plantations is scarce. Current papers are predominantly concerned with issues such as pollination, shade management, nanotechnology applications, roasting effects, disease management, and environmental impacts [14]. Accordingly, this study was carried out to evaluate the effect of varied amounts of nitrogen, phosphorus and potassium and the application of dolomitic lime on the yield of the Typica and Oro azteca coffee varieties.

## 2. Results

### 2.1. Soil Characterization

Soil samples taken from varied depths in the soil profile of both experimental sites were analyzed for chemical and physical properties (Table 1), according to the procedures described in the with the Mexican standard NOM-021-RECNAT-2000 [15]. Coarse loamy textures are common as a result of low weathering processes because of the high slope values. In accordance with texture, bulk density values were 1.35 g cm^−3^ or higher. pH was strongly acidic at Finca la Galera and moderately acidic (very close to strongly acidic) at Finca la Concordia. Accordingly, calcium carbonate content was very low.

The amount of organic matter is high only at the uppermost soil horizon. Deeper than 25 cm, organic matter content is low to very low. Nitrogen content has a similar behavior to organic matter content. Phosphorus content is also very low, and potassium levels varied from low to medium values.

According to the horizon description and soil laboratory results, soils at both sites were classified as Lithic Ustortents [16] with low pedogenic evolution, because of the topographic relief conditions prevailing at the study area. The geologic settings at the study area correspond to metamorphic geological material (schist and gneis) from the medium Proterozoic age (1500 million of years).

### 2.2. Plant Height

**Figure 1:**
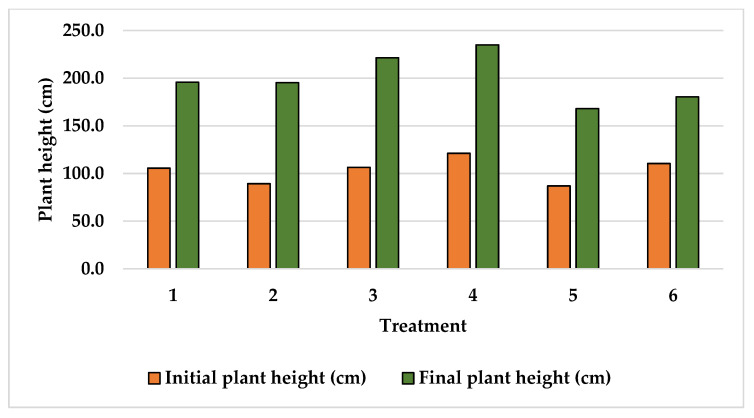
Initial and final plant height of the Typica variety at Finca la Concordia.

**Figure 2:**
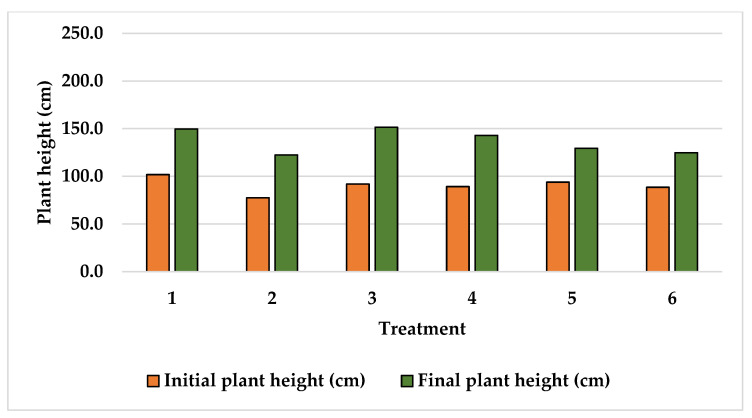
Initial and final plant height of the Oro azteca variety at Finca la Concordia.

**Figure 3:**
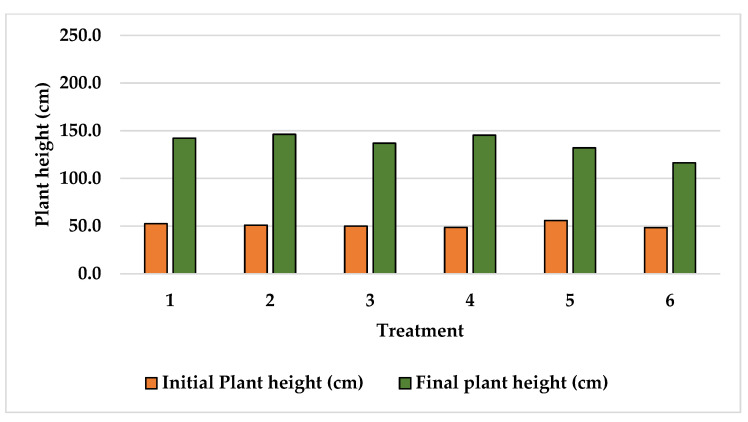
Initial and final plant height of the Oro azteca variety at Finca la Galera.

Plant height data recorded at the beginning and at the end of the experiments are shown in Figure 1, Figure 2 and Figure 3. The initial plant height recorded at Finca La Concordia of the four years old Typica variety plants averaged 103.3 cm with a variation coefficient of 12.6 while three years old Oro azteca variety plants averaged heights slightly lower at 90.5 cm with a variation coefficient of 8.8. The two-year-old Oro azteca variety at Finca La Galera registered the lowest initial plant height of 51.0 cm and a variation coefficient of 5.6. The final average plant heights at Finca La Concordia were as follows: Typica variety 199.3 cm with a variation coefficient of 12.5 and the Oro azteca variety averaged 136.8 cm with a variation coefficient of 9.4. The Oro azteca variety at Finca la Galera averaged a final plant height of 136.5 cm, with a variation coefficient of 8.2.

**Figure 4:**
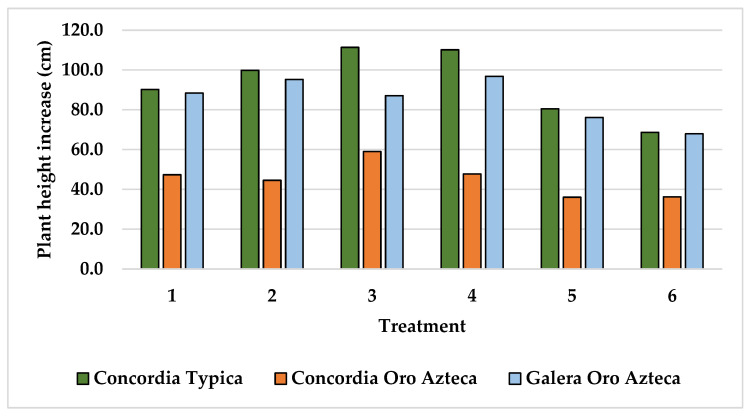
Plant height increments of the coffee plantations at the study areas.

The plant height increment between the initial and the last recording is shown at Figure 4. The height increment of the Typica variety at Finca La Concordia averaged 93.4 cm with a variation coefficient of 18.1. The Oro azteca variety at Finca La Concordia averaged the lowest height increment with 45.3 cm and a variation coefficient of 19.0. The Oro azteca variety at Finca La Galera averaged a plant height increment of 85.5 cm and a variation coefficient of 13.2.

The analysis of variance (ANOVA) performed to the height increment variable resulted in significant differences among treatments with very low significance values of 0.0022, 0.0034 and 0.0049 for the varieties Typica and Oro azteca at Finca la Concordia and the Oro azteca variety at Finca la Galera, respectively. Accordingly, the Tukey test for means comparison with an alpha value of 0.05 was practiced (Table 2). Both varieties at Finca la Concordia had similar response, with treatment 3 (100-46-60; N-P-K; lime) being different than treatments 5 (18-12-06; N-P-K; no lime) and 6 (00-00-00; N-P-K; no lime) with low NPK levels. On the other hand, height increments of treatments 2 (100-46-120; N-P-K; no lime) and 4 (100-46-60; N-P-K; no lime) were different than the height increment of treatment 6 for the Oro azteca at Finca la Galera.

**Figure 5:**
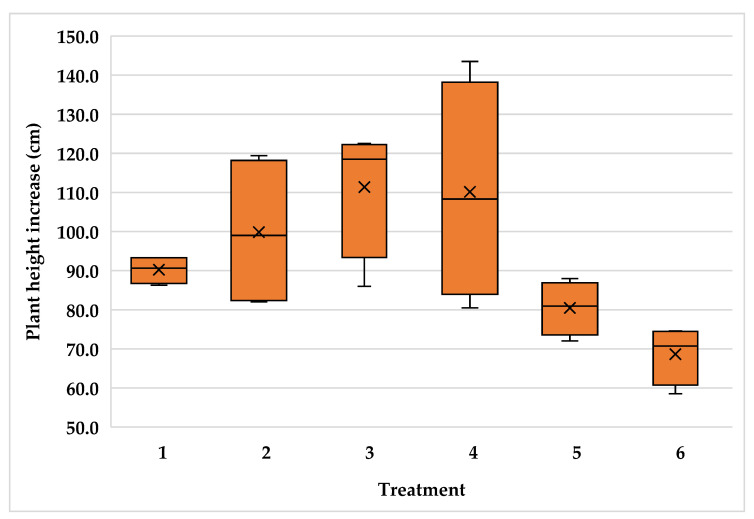
Plant height increment variability of the Typica variety at Finca la Concordia (Treatment´s mean, represented by X).

**Figure 6:**
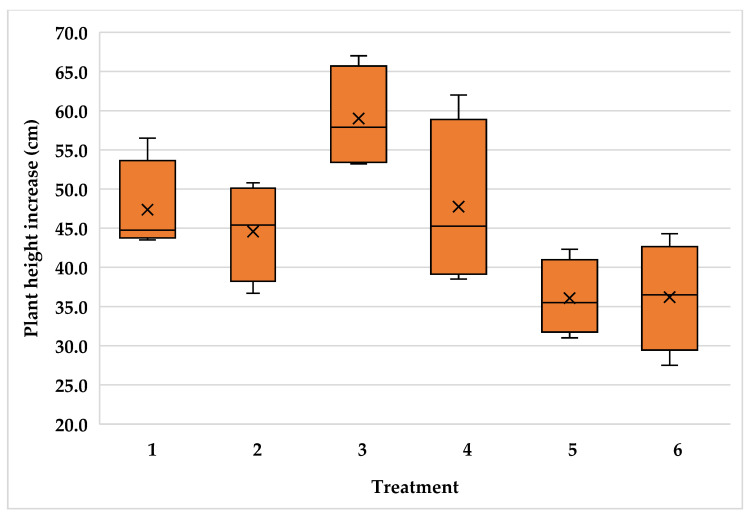
Plant height increment variability of the Oro azteca variety at Finca la Concordia (Treatment´s mean, represented by X).

**Figure 7:**
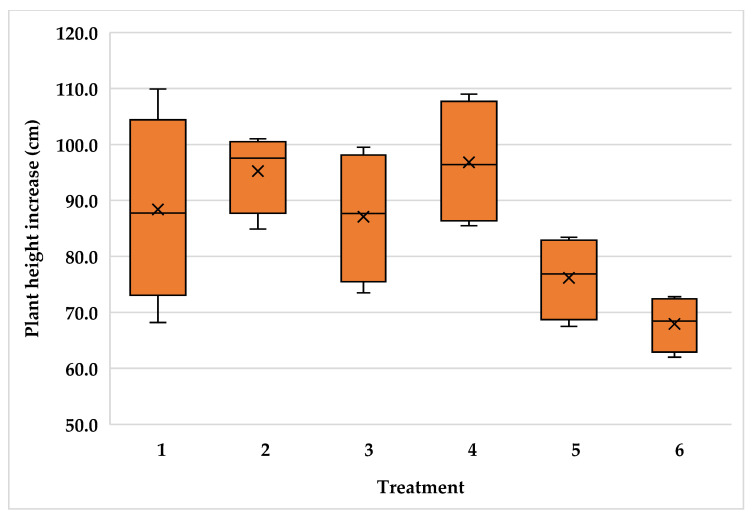
Plant height increment variability of the Oro azteca variety at Finca la Galera (Treatment´s mean, represented by X).

The high variability of plants inside the plantations resulted in high variability for plant height increment with variance coefficient values of 14.3, 15.7 and 11.3 for the Typica and Oro azteca varieties at Finca la Concordia and the Oro azteca variety at Finca la Galera, respectively. Figure 5, Figure 6 and Figure 7 show the minimum and maximum values, the 25, 50 and 75 percentiles and the average value for each treatment. These values show high range values mainly for treatments 2, 3 and 4 in the Typica variety and treatment 4 in the Oro azteca variety both at Finca la Concordia and treatments 1, 3 and 4 in the Oro azteca variety at Finca la Galera.

### 2.3. Coffee Yield

**Figure 8:**
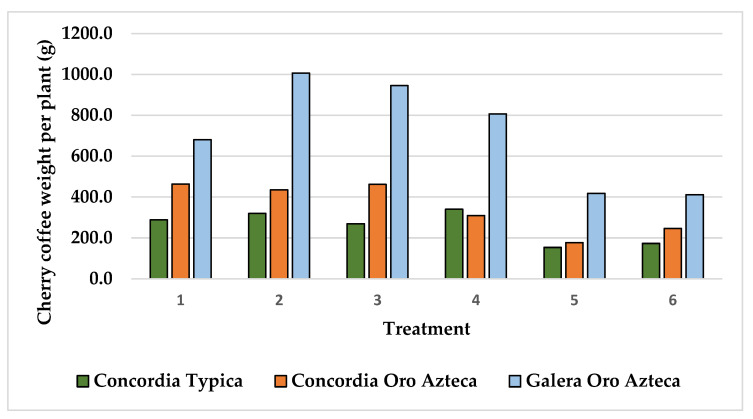
Cherry coffee weight per plant of the coffee plantations at the study area.

The experiments were harvested in January 2022, registering the weight of ripe cherry coffee grains for each coffee plant. Figure 8 shows that even when the Typica variety had been planted 8 years before the harvest time, the cherry coffee weight per plant was the lowest of all with an average of 257.1 g per plant. On the other side, the Oro azteca at Finca la Galera resulted in the highest ripe cherry coffee average with 711.1 g per plant, although this plantation had only been established for 6 years when harvested.

The analysis of variance (ANOVA) performed of the cherry coffee weight variable resulted in differences among treatments at very low significance values of 0.0031, 0.0002 and 0.0001 for the Typica and Oro azteca varieties at Finca la Concordia and the Oro azteca variety at Finca la Galera, respectively. Accordingly, the Tukey test for means comparison with an alpha value of 0.05 was practiced (Table 3). The three experiments showed that the treatments with low or zero NPK application (treatments 5 and 6) were statistically different than treatment 2 (100-46-120; N-P-K; no lime). On the other side, treatment 4 (100-46-60; N-P-K; no lime) was superior to treatments 5 and 6 at the Typica variety in Finca la Concordia and the Oro azteca variety in Finca la Galera, and treatment 3 was superior to treatments 5 and 6 at the Oro azteca variety for both sites. Treatment 1 was superior to treatments 5 and 6 only in the Oro azteca variety at Finca la Concordia.

**Figure 9:**
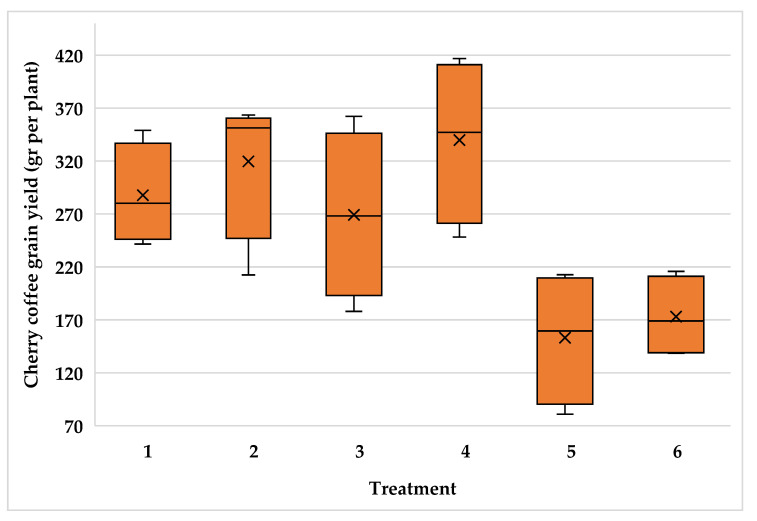
Cherry coffee yield variability of the Typica variety at Finca la Concordia (Treatment´s mean, represented by X).

**Figure 10:**
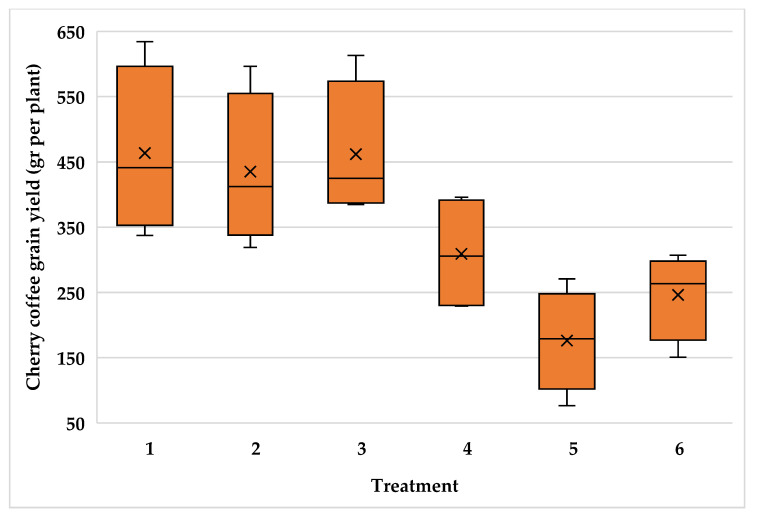
Cherry coffee yield variability of the Oro azteca variety at Finca la Concordia (Treatment´s mean, represented by X).

**Figure 11:**
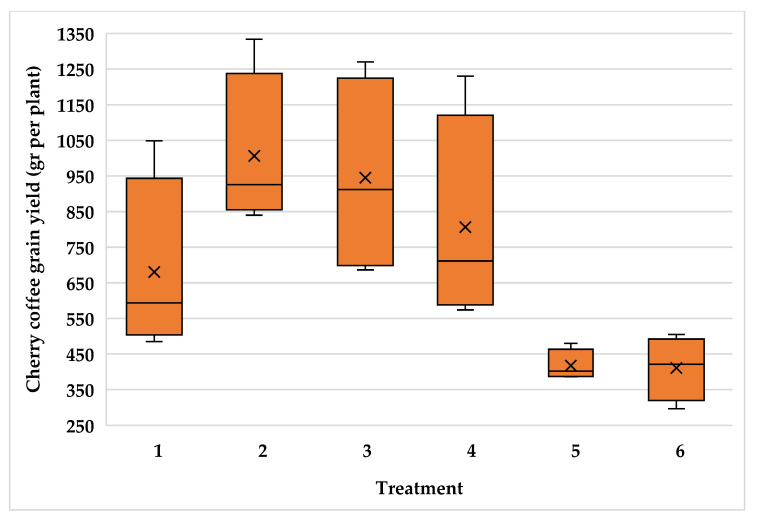
Cherry coffee yield variability of the Oro azteca variety at Finca la Galera (Treatment´s mean, represented by X).

The high variability of plants inside the plantations resulted in high variability for cherry coffee yield with variance coefficient values of 24.5, 22.1 and 21.2 for the Typica and Oro azteca varieties at Finca la Concordia and the Oro azteca variety at Finca la Galera, respectively. Figure 9, Figure 10 and Figure 11 show the minimum and maximum values, the 25, 50 and 75 percentiles and the average value for each treatment. These values show high range values mainly for treatments 2, 3 and 4 for the Typica variety and treatments 1, 2 and 3 for the Oro azteca variety at Finca la Concordia and treatments 1, 2, 3 and 4 for the Oro azteca variety at Finca la Galera.

The cherry coffee yield per plant was converted to cherry coffee yield per hectare by using a plant density of 3000 plants per hectare (Table 4). Treatments 4 and 2 are superior to control treatments 5 and 6 for the Typica variety at Finca la Concordia while treatments 1, 2 and 3 at the for the Oro azteca variety at Finca la Concordia and treatments 2, 3 and 4 for the Oro azteca variety at Finca la Galera overcame the control treatments 5 and 6.

## 3. Discussion

Soil conditions in the study area were characterized by very low amounts of organic matter, nitrogen, potassium and phosphorus after 26 cm depth at Finca la Concordia and after 25 cm depth at Finca la Galera. These low nutrient contents, high bulk density values (higher than 1.45 g cm^−3^) and coarse textures (sandy loam and loamy sand) are related to the high soil slope gradient [17] prevailing at both sites, resulting in a Lithic Ustortents soil with low pedogenic evolution.

The low nutrient content in the soils of the study area is one of the reasons for the low productivity of coffee plantations. Plant height increment at treatments with zero N, P and K application resulted in low values of 68.7, 36.2 and 67.9 cm for the Typica and Oro azteca varieties at Finca la Concordia and the Oro azteca variety at Finca la Galera, respectively. In accordance with plant height increment, cherry coffee yield per plant was also low for treatments with zero N, P and K application, with values of 173.1, 246.3, and 411.0 g per plant for the Typica and Oro azteca varieties at Finca la Concordia and the Oro azteca variety at Finca la Galera. These low values of coffee productivity are related to the low nutrition content prevailing in plantations in agroforestry systems with hilly terrain conditions [3].

These coffee yields per plant are equivalent to 0.52, 0.74, and 1.23 t ha^−1^ for the Typica and Oro azteca varieties at Finca la Concordia and the Oro azteca variety at Finca la Galera. These low yields are characteristic of southern Mexico, mainly in the Oaxaca coffee production area where an average yield of 0.77 t ha^−1^ of cherry coffee is reported, compared with 3.4 t ha^−1^ and of cherry coffee at the Mexican state of Puebla [5].

It is very important to recognize that agroforestry systems are very important in hilly areas where the lack of vegetation cover represents a high risk for soil erosion. Besides providing shade for coffee plants, the trees in an agroforestry system produce plant residue for the soil surface, protecting the soil from erosion under high slope conditions and increasing the soil organic matter [18,19,20].

On the other side, the application of N-P-K fertilizers resulted in 111.4, 59.0 and 87.1 cm of plant height increments registered for the Typica and Oro azteca varieties at Finca la Concordia and the Oro azteca variety at Finca la Galera for the treatment with application of 100, 46, and 60 kg ha^−1^ of N-P-K. These values contrasted with 319.7, 435.1 and 1006.3 g per plant registered for the same varieties and sites for the treatment with application of 100, 46, and 120 kg ha^−1^ of N-P-K, in accordance with the plant requirements [9,10].

The Typica variety evaluated at Finca la Concordia reached a yield of 1.02 t ha^−1^ with the application of 100-46-60 kg ha^−1^ of N-P-K, compared with 0.52 t ha^−1^ with the control treatment and 0.46 t ha^−1^ with the low N-P-K rate of 18-12-02 kg ha^−1^. The Oro azteca variety also had a good response to treatment 4 yielding 0.93 t ha^−1^ at Finca la Concordia and 2.42 t ha^−1^ at Finca la Galera. The main factor for the great yield difference between these two sites is better plantation management at Finca la Galera.

Nitrogen is the most important coffee nutrient with general recommended rates of 400 kg ha^−1^ for Brazil and 300 to 400 kg ha^−1^ for Colombia [21]. However, it is very important to make proper application considering factors such as source, rate, application timing and place because nitrogen loss by volatilization, denitrification and leaching may reach up to 50% of the applied fertilizer [22]. Nitrogen requirements in shade agroforestry systems are considerably lower than in monoculture systems mainly because of low plant densities.

Even though the Oro azteca variety produced yields higher than 1.3 t ha^−1^ with treatments 1, 2 and 3, the high variability of both, plant height increment and cherry coffee yield did not allow these treatments to be statistically different to treatment 4. For this reason and also because of the lower cost, treatment 4 is preferred. A similar situation appeared for the Oro azteca variety at Finca la Galera where treatments 2 (3.02 t ha^−1^) and 3 (2.84 t ha^−1^) resulted in higher yields of cherry coffee than treatment 4.

## 4. Materials and Methods

### 4.1. The Study Area

**Figure 12:**
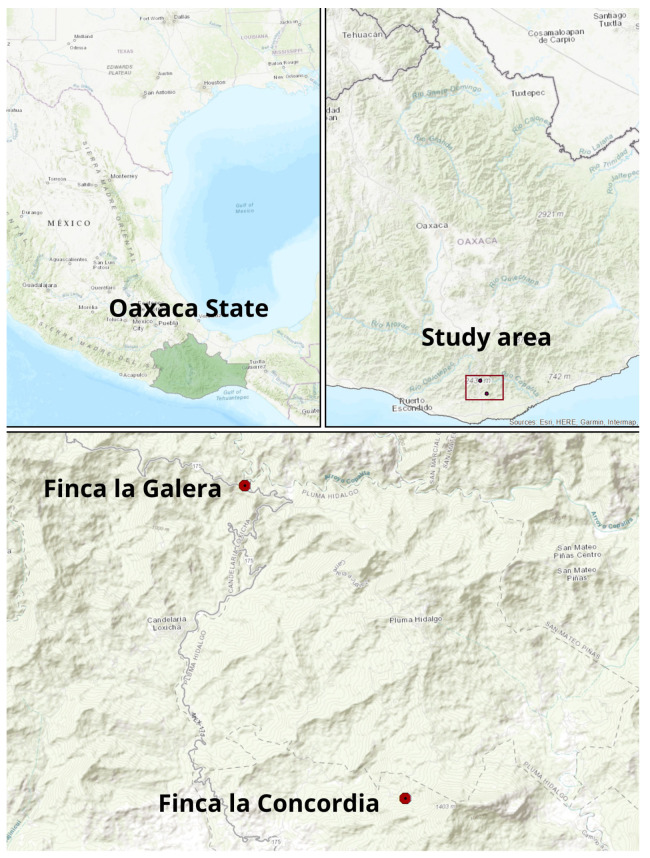
Location of the experimental sites.

The study was carried out at the coffee farms identified as “Finca la Concordia” and “Finca la Galera” in the municipalities of Candelaria Loxicha and San Pedro Pochutla, respectively. These municipalities are located in the state of Oaxaca at southern Mexico very close to the Pacific Ocean (Figure 12) and are characterized by having a warm subhumid climate, with summer rains (800 to 3500 mm) and a temperature range between 18 °C and 28 °C. Geographic location and altitude characteristics are shown in Table 5. Both sites are located on hilly terrain, as can be seen in Figure 12, with soil slope mostly higher than 50% and with the presence of trees that provide shade to the coffee plants.

A soil profile was described [23] on each of the farms, and soil samples were taken from each identified horizon for the determination of physical and chemical properties in accordance with the Mexican standard NOM-021-RECNAT-2000 [15]. Soil analyses were carried out based on the methodology established by Van Reeuwijk [24] considering the following physical properties: bulk density (g cm^−3^) and texture class. The chemical properties were pH; organic matter (%); nitrogen (%); potassium (Cmol(+) Kg^−1^); phosphorus (Mg Kg^−1^) and calcium carbonates (%).

### 4.2. Description of the Experiments

Two experiments were carried out at the site identified as “Finca la Concordia” with the coffee variety known as “Typica” in one experiment and the “Oro azteca” variety at the other experiment. When the experiments were established in August 2018, the “Oro azteca” variety was three years old and the Typica variety was four years old. Another experiment was established at the site identified as “Finca la Galera” where the Oro azteca variety had two-year-old plants when the experiment was established.

The Typica variety evolved from the original coffee variety introduced in Oaxaca in the nineteenth century in response to the local environmental conditions and is highly appreciated because of its good cup quality [25] but is highly susceptible to the coffee leaf rust. The Oro azteca variety was released in Mexico as a product of breeding activities between the hybrid Timor with leaf rust resistance and the variety Caturra Rojo with good yield and quality characteristics [26]. The Oro azteca variety has a lower height than the Typica variety, allowing plantation higher densities and, as a consequence, higher yields [27].

The experimental design was a randomized complete block design with six treatments (Table 6) and four replications. The treatments were selected according to varied amounts of N, P, K and lime. Five treatments had N, P and K applications and the sixth treatment had no application of N-P-K, acting as a control treatment. The number of N-P-K treatments per plant was adjusted to a plant density of 3000 plants per hectare. Fertilizers used were urea (46% N), diammonium phosphate or DAP (18% N and 46% P_2_O_5_) and potassium chloride (60% K_2_O). The amount of dolomitic lime was 1.8 t ha^−1^, applied in August 2028, and after that the application was yearly in the month of July. The experimental unit consisted of six plants of the Typica variety at Finca la Concordia and four plants of the Oro azteca variety at both Finca la Concordia and Finca la Galera. At the beginning (August 2018) and at the end (August 2019) of the experiment, the height of the plants from the soil surface to the plant apex was registered. With these data, plant height increment between the beginning and the end of the experiment was obtained. Harvest of experiments was carried out in January 2022, and data about coffee cherry grain weight per harvested plant were registered for the evaluation of the treatments’ effect on coffee yield.

Data on plant height increment and cherry coffee grain yield were subjected to analyses of variance with the Statistical Analysis System (SAS) version 9.4 [28], and when significant difference among treatments appeared, Tukey means comparison at 5% probability was practiced.

## 5. Conclusions

The soil at the study area is a Lithic Ustortents soil with low pedogenic evolution, resulting in very low amounts of organic matter, nitrogen, potassium and phosphorus after 26 cm depth.

Coffee yield was low with zero N, P and K application resulting in 0.52, 0.74, and 1.23 t ha^−1^ for the Typica and Oro azteca varieties at Finca la Concordia and the Oro azteca variety at Finca la Galera.

The application 100, 46, and 120 kg ha^−1^ of N-P-K resulted in coffee yields of 0.96, 1.31 and 3.02 t ha^−1^ for the Typica and Oro azteca varieties at Finca la Concordia and the Oro azteca variety at Finca la Galera.

The Typica variety evaluated at Finca la Concordia reached a yield of 1.02 t ha^−1^ with the application of 100-46-60 kg ha^−1^ of N-P-K, compared with 0.52 t ha^−1^ with the control treatment and 0.46 t ha^−1^ with the low N-P-K rate. The Oro azteca variety also had a good response to this fertilization rate yielding 0.93 t ha^−1^ at Finca la Concordia and 2.42 t ha^−1^ at Finca la Galera.

With the yearly application of N-P-K rates of 100-46-60 kg ha^−1^ in agroforestry coffee plantations in the Oaxaca’s Coastal coffee producing region, a yield of 1.0 t ha^−1^ for the Typica variety and 1.5 t ha^−1^ for the Oro azteca variety is expected.

Proper application of nitrogen fertilizers must consider time of application and covering the fertilizer with soil in order to avoid volatilization loss. In order for plant nutrition to be effective good management practices such as pruning and weed control must be ensured. Organic nutrition practices must be implemented along with the inorganic materials in order to obtain medium- and long-term soil fertility.

## Figures and Tables

**Table 1:** Chemical and physical soil properties at experimental sites.

Depth (cm)	BD	Texture	pH	O.M.	N	K	P_2_O_5_	CaCO_3_
**Finca la Concordia**								
0–5	1.37	Sandy loam	5.3	4.12	0.17	0.50	1.48	0.23
5–26	1.45	Sandy loam	5.3	3.99	0.13	0.23	0.00	0.75
26–50	1.52	Sandy loam	5.4	2.83	0.08	0.15	0.00	1.16
**Finca la Galera**								
0–25	1.35	Loamy sand	4.8	5.40	0.18	0.60	1.18	0.52
25–55	1.53	Loamy sand	4.7	0.90	0.04	0.39	0.00	1.04
55–83	1.75	Loamy sand	4.7	0.64	0.01	0.25	0.00	1.04
83–95	1.67	Loamy sand	4.6	0.39	0.00	0.25	0.00	1.1
95–115	1.35	Loamy sand	4.2	0.51	0.01	0.26	0.00	0.98

BD = bulk density (g cm^−3^) pH = hydrogen potential (H_2_O); O.M.= organic matter (%); N = total nitrogen (%); K = potassium (Cmol_(+)_ Kg^−1^); P_2_O_5_ = phosphorus (mg Kg^−1^); CaCO_3_ = equivalent calcium carbonate (%).

**Table 2:** Coffee plant height increment (cm) between August 2018 and August 2020.

Treatment	Finca la Concordia		Finca la Galera
	Typica Variety	Oro Azteca Variety	Oro Azteca Variety
1	90.2 abc	47.4 ab	88.4 ab
2	99.9 ab	44.6 ab	95.3 a
3	111.4 a	59.0 a	87.1 ab
4	110.2 ab	47.8 ab	96.8 a
5	80.5 bc	36.1 b	76.2 ab
6	68.7 c	36.2 b	67.9 b

Means with different lowercase letter are different by Tukey test (α = 0.05).

**Table 3:** Weight of cherry coffee per plant (g).

Treatment	Finca la Concordia		Finca la Galera
	Typica Variety	Oro Azteca Variety	Oro Azteca Variety
1	287.7 ab	463.5 a	680.3 ab
2	319.7 a	435.1 a	1006.3 a
3	269.2 ab	461.8 a	945.0 a
4	339.9 a	309.1 ab	806.6 a
5	153.2 b	176.4 b	417.5 b
6	173.1 b	246.3 b	411.0 b

Means with different lowercase letter are different by Tukey test (α = 0.05).

**Table 4:** Cherry coffee yield (t ha^−1^) with 3000 plants per hectare.

Treatment	Finca la Concordia		Finca la Galera
	Typica Variety	Oro Azteca Variety	Oro Azteca Variety
1	0.86 ab	1.39 a	2.04 ab
2	0.96 a	1.31 a	3.02 a
3	0.81 ab	1.39 a	2.84 a
4	1.02 a	0.93 ab	2.42 a
5	0.46 b	0.53 b	1.25 b
6	0.52 b	0.74 b	1.23 b

Means with different lowercase letter are different by Tukey test (α = 0.05).

**Table 5:** Geographic location of the experimental sites.

Farm	Longitude (West)	Latitude (North)	Altitude (masl)	Municipality
La Concordia	−96.42527	15.87399	741	San Pedro Pochutla
La Galera	−96.47062	15.96836	1160	Candelaria Loxicha

**Table 6:** Amounts (kg ha^−1^) of NPK and lime for the evaluated treatments.

Treatment	N	P_2_O_5_	K_2_O	Lime (1.8 t ha^−1^)
1	100	46	120	Yes
2	100	46	120	No
3	100	46	60	Yes
4	100	46	60	No
5	18	12	06	No
6 (Control)	0	0	0	No

## Data Availability

Data are contained within the article.
